# Detection of a novel reassortant epizootic hemorrhagic disease virus serotype 6 in cattle in Trinidad, West Indies, containing nine RNA segments derived from exotic EHDV strains with an Australian origin

**DOI:** 10.1016/j.meegid.2019.103931

**Published:** 2019-10

**Authors:** Paulina Rajko-Nenow, Tamiko Brown-Joseph, Chandana Tennakoon, John Flannery, Christopher A.L. Oura, Carrie Batten

**Affiliations:** a Non-vesicular reference laboratory, The Pirbright Institute, Woking, Surrey GU24 0NF, UK; b Integrative Biology & Bioinformatics, The Pirbright Institute, Woking, Surrey GU24 0NF, UK; c School of Veterinary Medicine, Faculty of Medical Sciences, The University of the West Indies, St. Augustine, Trinidad and Tobago

**Keywords:** EHDV, Trinidad, Cattle, Reassortment, Next generation sequencing

## Abstract

Epizootic hemorrhagic disease virus (EHDV) is a *Culicoides*-transmitted orbivirus that infects domestic and wild ruminants in many parts of the world. Of the eight proposed serotypes, only EHDV-1, 2 and 6 have been reported to be present in the Americas. Following the identification of a virulent EHD-6 reasssortant virus in the USA in 2007 (EHDV-6 Indiana), with outer coat protein segments derived from an Australian strain of EHDV and all remaining segments derived from a locally circulating EHDV-2 strain, questions have remained about the origin of the Australian parent strain and how it may have arrived in the USA. When EHDV-6 was identified in asymptomatic cattle imported into the Caribbean island of Trinidad in 2013, full genome sequencing was carried out to further characterise the virus. The EHDV-6 Trinidad was a reassortant virus, with 8 of its 10 segments, being derived from the same exotic Australian EHDV-6 strain as the VP2 and VP5 present in the EHDV-6 Indiana strain from the USA. Analyses of the two remaining segments revealed that segment 8 showed the highest nucleotide identity (90.4%) with a USA New Jersey strain of EHDV-1, whereas segment 4 had the highest nucleotide identity (96.5%) with an Australian EHDV-2 strain. This data strongly suggests that the Trinidad EHDV-6 has an Australian origin, receiving its segment 4 from a reassortment event with an EHDV-2 also from Australia. This reassortant virus likely came to the Americas, where it received its segment 8 from a locally-circulating (as yet unknown) EHDV strain. This virus then may have gained entry into the USA, where it further reassorted with a known locally-circulating EHDV-2, the resulting strain being EHDV-6 Indiana. This study therefore identifies, for the first time, the likely minor parent virus of the EHDV-6 currently circulating in the USA.

## Highlights

• A unique reassortant EHD-6 was circulating asymptomatically in Trinidad in 2013
• Nine of the ten segments making up the EHD-6 Trinidad virus had a likely Australian origin
• The EHDV-6 Trinidad is likely to be the minor parent virus of the EHDV-6 Indiana strain currently circulating in the USA.

## 1 Introduction

Epizootic hemorrhagic disease virus (EHDV) is a *Culicoides*-transmitted virus that causes a severe haemorrhagic disease with high morbidity and mortality in white-tailed deer in North America. In contrast, mule deer, black-tailed deer and pronghorn antelope are less affected by the virus, having higher survival rates (Savini et al., 2011). EHDV had rarely been associated with clinical disease in domestic cattle in the past decades, with the exception of Ibaraki virus which caused a large-scale epizootic in Japan in 1959 (Omori et al., 1969). More recently however, after reports of severe clinical outbreaks of epizootic hemorrhagic disease (EHD) in dairy and beef cattle from Turkey, Morocco, Tunisia, Algeria, Israel and Jordan, EHD was added to the list of notifiable diseases by the World Organization for Animal Health (OIE) (EFSA, 2009).

EHDV belongs to the genus Orbivirus within the family *Reoviridae* and is closely related to Bluetongue virus (BTV). The genomes of both viruses comprise ten segments of linear double-stranded (ds) RNA, which encode for seven structural (VP1-VP7) and non-structural proteins (NS1-NS4 and possibly NS5) (Anthony et al., 2009a; Mecham and Dean, 1988; Stewart et al., 2015). Structural proteins VP2 and VP5 comprise the outer capsid layer and are mainly responsible for receptor-binding and penetration of host cells (Anthony et al., 2009b). Virus serotype is primarily determined through the specificity of VP2 interactions with neutralizing antibodies; however, VP5 may also contribute to the specificity of the neutralizing antibody response (Anthony et al., 2009b). Based on phylogenetic analysis and neutralisation tests, eight distinct EHDV serotypes have been proposed, with the most-recently characterised serotype being reported in asymptomatic cattle in Japan (Shirafuji et al., 2017). EHDV serotypes can be clustered into four distinct groups (A-D), which have been shown to correspond well with serological properties of the virus (Anthony et al., 2009b), with no cross-neutralisation occurring between the groups.

Similar to other Orbiviruses, EHDV evolution is largely driven by two main forces; random mutation and segment reassortment. The former occurs during natural transmission cycles and plays an important role in the diversification of EHDV strains and their pathogenicity. A small number of nucleotide substitutions can have an impact on overall pathogenicity as has been shown for BTV serotype 8 (BTV-8) (Flannery et al., 2019). Segment reassortment is a consequence of segment exchange, when cells are co-infected with at least two different EHDV strains.

In 2006, a novel reassortant strain of EHDV-6 (Indiana) was detected in the USA, in which segments 2 and 6 originated from an Australian virus (EHD6/AUS1981/07 known also as CSIRO 753) and the remaining 8 segments originated from a North American EHDV-2 strain from Alberta (Allison et al., 2010). Following the identification of EHDV-6 Indiana (Anbalagan et al., 2014), a sudden increase of disease caused by EHDV-6 was reported across Nebraska, South Dakota, Michigan and Missouri in domestic cattle and white-tailed deer (Stevens et al., 2015).

In 2013, a group of EHDV-naive cattle imported from the USA onto the island of Trinidad, seroconverted for EHDV antibodies within six months of their arrival on the island (Brown-Joseph et al., 2019). The detection of EHDV RNA in the cattle in the absence of clinical signs indicated an asymptomatic infection in these animals. EHDV segment-2 sequence analysis revealed that the Trinidad 2013 EHDV-6 VP2 sequence was very similar to the EHDV-6 VP2 sequences in strains from in Guadalupe (2010), Martinique (2010), USA (2006) and Australia (1981), with 96–97.2% nucleotide identity.

The objective of this study was to perform full genome sequencing on the Trinidadian EHDV-6 isolate, in order to identify the degree of reassortment within the virus. Phylogenetic sequence comparison of each segment would then enable conclusions to be made about the likely provenance of each segment of the virus, giving clues to how the virus may have evolved, and how it may be related to the EHDV-6 strains currently circulating and causing severe disease in the USA.

## 2 Material and methods

### 2.1 Study background

In 2013, sixty Holstein and Jersey dairy cattle were imported into Trinidad & Tobago from the USA. Upon arrival in Trinidad, all animals (*n* = 60) were confirmed as EHDV RNA negative using the EHDV real-time PCR assay (Brown-Joseph et al., 2019). All naïve animals seroconverted within 6 months of their arrival in the country, indicating that they were infected with EHDV while in Trinidad. No animals showed clinical signs consistent with EHD throughout the six-month period (Brown-Joseph et al., 2019).

### 2.2 Sample selection and virus isolation

Blood samples were collected from cattle monthly over a six-month period and were tested using a group-specific real-time RT-PCR assay targeting EHDV segment 9 (Maan et al., 2017). EHDV-positive blood samples tested positive for serotype 6 (EHDV-6) and negative for other serotypes: EHDV-1, EHDV-2, EHDV-4, EHDV-5, EHDV-7, EHDV-8 and EHDV-9 (Brown-Joseph et al., 2019). EHDV-6 was isolated in KC cells (derived from embryonic *Culicoides sonorensis)* from a blood sample, collected from a Jersey cow, two months after its arrival into Trinidad. This isolate, named as TAT2013/02 [KC2], was deposited in The Pirbright Institute, Orbivirus Reference Collection and is available through the European Virus Archive goes global catalogue (https://www.european-virus-archive.com/evag-portal). TAT2013/02 isolate was repassaged two more times in KC cells as previously described (Batten et al., 2011), to increase the viral load. A C_T_ value of <12 was confirmed using the EHDV group-specific real-time RT-PCR. Passage TAT2013/02 [KC4] was then selected for sequencing.

### 2.3 Next generation sequencing

Total RNA was extracted from the cell culture pellet using TRIzol Reagent (Life technologies, UK) and ssRNA was removed by precipitation in 2 M lithium chloride (Sigma, UK) overnight as described (Maan et al., 2007). The dsRNA (8 μl) was denatured by heating at 95 °C for 5 min and the first cDNA strand was synthesised using SuperScript III RT (Life technologies, UK) and then the second strand was synthesised using NEBNext (New England BioLabs, UK) according to the manufacturers' instructions. Double stranded (ds) cDNA was purified using the Illustra GFX PCR DNA and Gel Band Purification kit (GE Healthcare, UK) and quantified with the Qubit dsDNA HS Assay kit (Life technologies, UK). The concentration of dscDNA was then adjusted to 0.2 ng/μl with 10 mM Tris-HCl, pH 8.0 buffer. Libraries were prepared using the Nextera XT library preparation kit and sequencing was performed using MiSeq Reagent kit v2 (Illumina, USA) on the MiSeq benchtop sequencer.

### 2.4 Genome assembly

A pre-alignment quality check was performed using the FASTQC programme and the Trim Galore programme was used for adapter trimming and quality trimming of reads at the Phred quality threshold of 30 and removal of short reads (<50 bp). Subsequently, reads were aligned to the reference genome (EHD6/AUS1981/07 virus) for segments 1,2,3,4,5,6,7,9, and 10, and for segment 8 to the EHD1/USA/1995/01 virus sequence (known also as the New Jersey strain), using the BWA-MEM tool and a combination of samtools (mpileup) and bcftools to derive the consensus sequence (GenBank accession numbers: MK919254-MK919263).

### 2.5 Reassortment analysis

A number of full genome EHDV sequences (*n* = 51 on 1/12/2018) are publicly available through GenBank. In order to save computation time, 30 full-genome reference sequences were selected to represent all EHDV serotypes distributed worldwide and were retrieved from GenBank (Supplementary material Table 1). Amino acid sequences for each coding region (VP1, VP2, VP3, VP4, NS1, VP5, VP7, NS2, VP6, NS3) were aligned individually using the Muscle algorithm. The evolutionary distances between a pair of nucleotide sequences were calculated separately for each coding region using the Maximum Composite Likelihood Model (Tamura et al., 2004) in MEGA6 program (Tamura et al., 2013). All positions containing gaps were excluded from the calculations. Reassortment analysis was performed using Recombinant Detection Program version 4.95 (RDP4) (Martin et al., 2015) under default settings. Briefly, all EHDV coding regions were combined using an in-house biopython script for each previously selected reference strain (*n* = 30) and the EHD6/Trinidad/2013 virus, then full coding region were aligned on the amino acid level. The detection of potential recombination events was performed with the following methods: RDP, GENECONV, Maximum Chi Square, CHIMAERA, BOOTSCANing, Sister Scanning (SISCAN) and 3SEQ. Sequences were considered as reassortants if they were identified by at least by 5 recombinant detection methods. For graphical representation of the relationship between the possible reassortant virus (EHD6/Trinidad/2013) and its potential parental sequences, the distance plot was constructed using a window size of 200, a step size of 20 and the similarity model in the RDP4.

### 2.6 Phylogenetic analysis (VP2, VP4, VP5 and NS2)

Multiple sequence alignment was performed using the Muscle algorithm of the MEGA6 software and phylogenetic trees were reconstructed using IQ-Tree software version 1.3.11.1 (Nguyen et al., 2015). The best-fit model of evolution was selected according to the Bayesian information criterion score calculated using the IQ-Tree software. The maximum likelihood tree was based on the GTR + I + G4 model of substitution for the VP2 region, the TN + I model of substitution for VP4, the GTR + G4 model of substitution for VP5 and on the TN + I + G4 model of substitution for NS2. The reliability of the trees was estimated by ultrafast bootstrap (Minh et al., 2013) analysis of 1000 replicates and the clade was considered to be supported at a value ≥95%. All phylogenetic trees were visualised and rooted on the midpoint in MEGA6 software.

## 3 Results

Estimates of evolutionary distance between EHD6/Trinidad/2013 virus and each of the thirty selected reference strains are shown in Table 2. The lowest value of evolutionary divergence was found between viruses EHD6/AUS1981/07 and EHD6/Trinidad/2013 for almost all proteins, including VP1, VP2, VP3, NS1, VP5, VP7, NS2, VP6 and NS3, indicating that EHD6/Trinidad/2013 virus is most closely related to the EHD6/AUS1981/07 virus. For the remaining proteins, VP4 and NS2, the lowest value of evolutionary divergence was estimated as 0.0360 between EHD6/Trinidad/2013 and EHD2/AUS1979/05 and as 0.1733 between EHD6/Trinidad/2013 and two strains, namely EHD1/USA1955/01 and EHD1/USA/1974.

**Table 2:** Estimates of Evolutionary Divergence between the EHD6/Trinidad/2013 strain and the EHDV reference strain for different coding regions.

Protein name	VP1	VP2	VP3	VP4	NS1	VP5	VP7	NS2	VP6	NS3
Segment	seq-1	seg-2	seg-3	seg-4	seg-5	seg-6	seg-7	seg-8	seg-9	seg-10
Length of the sequence compared (nt)	3909	2964	2700	1935	1656	1587	1050	1122	1012	687
EHD6_AUS1981/07	0.0213*	0.0092*	0.0234*	0.0796	0.0252*	0.0251*	0.0235*	1.2255	0.0271*	0.0096*
EHD2_AUS1979/05	0.1266	1.5839	0.0555	0.0360*	0.0605	0.4773	0.2686	1.2261	0.0701	0.0211
EHD5_AUS1977/01	0.0330	1.5029	0.0291	0.0849	0.0340	0.6603	0.2801	1.2646	0.0292	0.0156
EHD7_AUS1981/06	0.0381	1.5920	0.0499	0.0693	0.0334	0.4701	0.2584	1.2611	0.0521	0.1297
EHD8_AUS1982/06	0.0668	0.6192	0.0828	0.0841	0.1570	0.1507	0.2824	1.3827	0.0754	0.1211
EHD6_BAR1983/01	0.2758	0.5112	0.2496	0.3195	0.2521	0.2452	0.2954	0.3377	0.4112	0.1335
EHD2_CAN1962/01	0.2873	1.6165	0.2436	0.3219	0.2422	0.4862	0.2924	0.1838	0.3952	0.1233
EHD7_ISR2006/02	0.2789	1.5817	0.2436	0.3204	0.2558	0.4753	0.2575	0.3335	0.3965	0.1321
EHD2_JAP1959/01	0.0584	1.6048	0.0725	0.0717	0.0685	0.4794	0.2691	1.0733	0.1212	0.0454
EHD1_NIG1967/01	0.2789	1.5089	0.2424	0.3199	0.2509	0.6312	0.2967	0.1904	0.4035	0.1146
EHD4_NIG1968/01	0.2825	1.5237	0.2395	0.3114	0.2531	0.6368	0.3211	0.5150	0.4045	0.1082
EHD6_South_Africa_1996	0.2738	0.5132	0.2398	0.3131	0.2568	0.2511	0.3001	0.5326	0.3978	0.1391
EHD1_USA1955/01	0.2842	1.4969	0.2450	0.3244	0.2423	0.6132	0.2988	0.1733*	0.3960	0.1306
EHD1_USA_1974	0.2850	1.4962	0.2457	0.3260	0.2422	0.6099	0.2988	0.1733*	0.3956	0.1315
EHD1_USA_1972	0.2842	1.4962	0.2456	0.3260	0.2422	0.6099	0.2988	0.1752	0.3993	0.1315
EHD1_USA_2006	0.2888	1.5077	0.2399	0.3205	0.2496	0.6048	0.3003	0.1926	0.3926	0.1299
EHD1_USA_2008	0.2877	1.5086	0.2400	0.3204	0.2435	0.6393	0.2956	0.1826	0.3956	0.1251
EHD1_USA_2010	0.2910	1.5082	0.2464	0.3245	0.2459	0.6192	0.3020	0.1958	0.3947	0.1261
EHD1_USA_2015	0.2879	1.5154	0.2475	0.3254	0.2502	0.6155	0.3000	0.1942	0.3994	0.1307
EHD2_USA_1971	0.2855	1.6190	0.2490	0.3245	0.2420	0.4884	0.2854	0.1875	0.3886	0.1242
EHD2_USA_1975	0.2880	1.6191	0.2414	0.3243	0.2413	0.4932	0.2954	0.1875	0.3943	0.1251
EHD2_USA_1990	0.2874	1.6137	0.2399	0.3214	0.2423	0.4840	0.2850	0.1839	0.3939	0.1279
EHD2_USA_1993	0.2874	1.6157	0.2404	0.3214	0.2431	0.4848	0.2850	0.1839	0.3956	0.1269
EHD2_USA_2004	0.2920	1.6297	0.2441	0.3219	0.2483	0.4920	0.2938	0.1901	0.3902	0.1279
EHD2_USA_2012	0.2904	1.6168	0.2416	0.3207	0.2388	0.4795	0.2923	0.2199	0.4027	0.1242
EHD2_USA_2013	0.2921	1.6200	0.2420	0.3230	0.2387	0.4791	0.2901	0.2202	0.3972	0.1251
EHD2_USA_2015	0.2920	1.6201	0.2420	0.3243	0.2363	0.4820	0.2917	0.2202	0.3993	0.1262
EHD6_USA_2006	0.2888	0.0116	0.2426	0.3231	0.2390	0.0291	0.2882	0.2058	0.3889	0.1251
EHD6_USA_2012	0.2882	0.0118	0.2458	0.3188	0.2435	0.0331	0.2821	0.2165	0.3977	0.1270
EHD6_USA_2016	0.2862	0.0138	0.2473	0.3252	0.2452	0.0311	0.2890	0.2371	0.3947	0.1261

The pair of nucleotide sequences with the lowest evolutionary distance calculated was indicated by (*) for each segment.

**Fig. 1:**
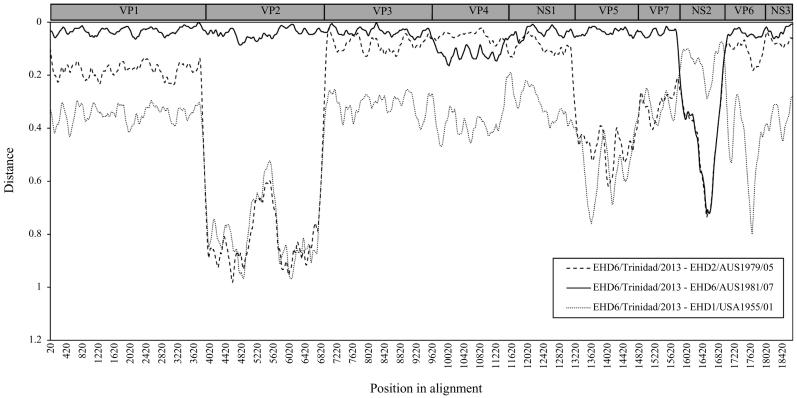
Distance plot constructed using RDP one query sequence (EHD6/Trinidad/2013) and three other sequences (EHD2/AUS/1979/05, EHD6/AUS1981/07, and EHD1/USA1955/01) based on the similarity model, window size of 200, and step size of 20.

Two recombination events were identified for the EHD6/Trinidad/2013 virus and were supported by at least 5 different recombinant detection methods. For the first recombination event, the beginning and end breakpoint positions were estimated at 9664 (99% Cl: 9415–9794) and 11,470 (99% Cl:11359–11,582). These positions correspond to nucleotides 9574–11,508 encoding for the minor structural protein VP4. The beginning and end breakpoint positions of the second recombinant event were estimated at a location of 15,844 (99% Cl: 15791–15,881) and 17,090 (99% Cl: 16935–17,153), which corresponds to the NS2 coding region. The genomic exchange of the NS2 and VP4 regions between EHD6/Trinidad/2013 virus and the parental sequences was confirmed by six and five different detection methods, respectively (Table 3). The major parental sequence was identified as EHD6/AUS1981/07 virus for both events, which is in-line with the estimates of evolutionary distances analysis (Table 1). Under the RDP analysis of recombination, EHD2/AUS1979/05 virus was recognised as the minor parental sequence for the VP4 coding region, whereas the minor parent for the NS2 region could not be assigned. Based on the evolutionary distance analysis (Table 2), the EHD1/USA1955/01 NS2 sequence showed the highest nucleotide identity (90.4%) with the EHD6/Trinidad/2013 NS2, indicating that this virus is the closest relative for the NS2 coding region. However, taking into consideration the relatively low level of identity (90.4%), there may well be another unknown EHD circulating in the Americas that is the minor parent for NS2. The genetic relationship between the reassortant EHD6/Trinidad/2013 virus and its potential parental sequences is graphically represented by the distance plot constructed based on the similarity model (Fig. 1). The nucleotide identity between EHD6/Trinidad/2013 virus and EHD6/AUS/1981/07 virus was calculated as 97.2% and 97.5% for segments 2 and 6 respectively, and 92.4% and 69.8% for segments 4 and 8 respectively. For segment 4 the highest nucleic acid identity was estimated between viruses EHD6/Trinidad/2013 and EHD2/AUS/1979/05 (96.5%), whereas for segment 8 the highest nucleotide identity was estimated as 90.37% between EHD6/Trinidad/2013 virus and each of the following strains: EHD1/USA/1955/01, EHD1/USA/1972 and EHD1/USA/1974.

**Table 3:** Recombination events with the approximate breakpoint positions and mean *P* values for different recombination detection methods.

	Breakpoint positions in alignment		Parental sequence		Average P value for detection methods						
Possible reassortant sequence	Beginning	End	Minor	Major	RDP	GENECONV	Bootscan	Maxchi	Chimaera	SiSscan	3Seq
EHD6/Trinidad/2013	15,844 (corresponds to the beginning of NS2)	17,090 (corresponds to the end of NS2)	Unknown	EHD6/AUS1981/07	NS	1.37E-119	6.25E-114	4.18E-38	8.14E-38	1.04E-33	2.00E-12
EHD6/Trinidad/2013	9664 (corresponds to the beginning of VP4)	11,470 (corresponds to the end of VP4)	EHD2/AUS1979/05	EHD6/AUS1981/07	NS	3.44E-05	2.81E-18	7.35E-13	1.90E-14	7.62E-11	NS

NS, not significant.

### 3.1 Phylogenetic analysis

**Fig. 2:**
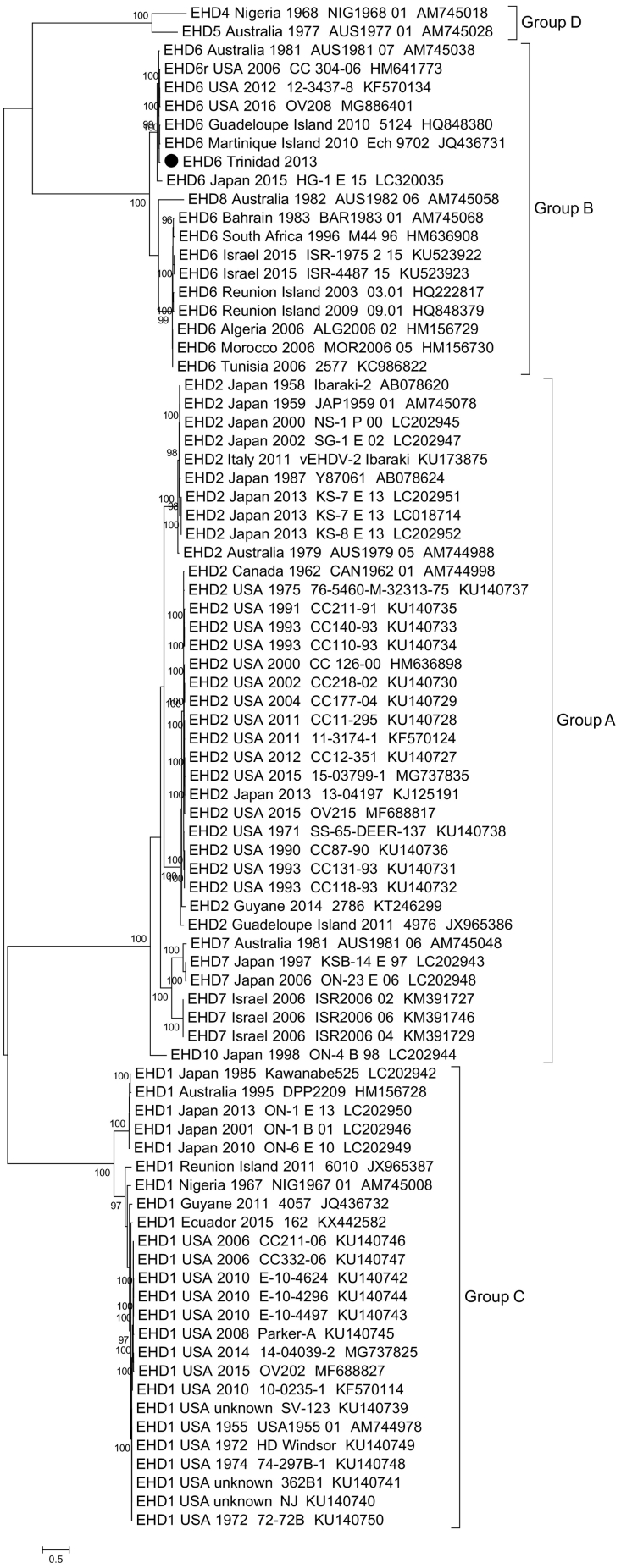

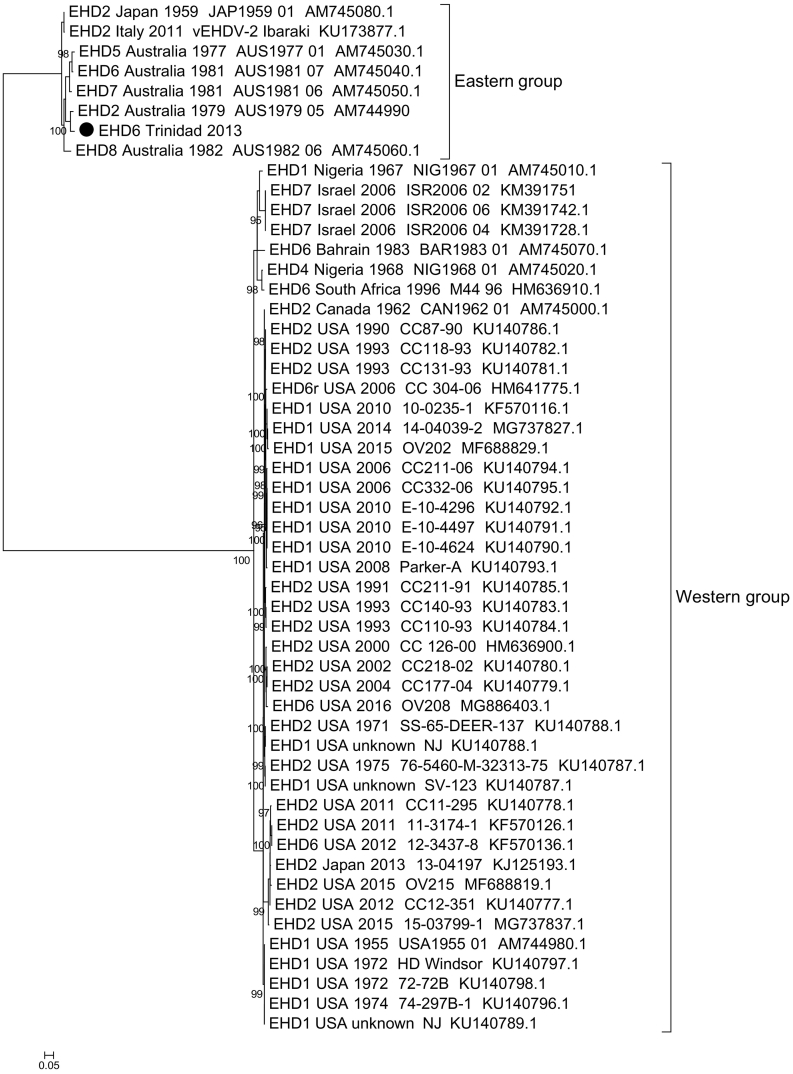

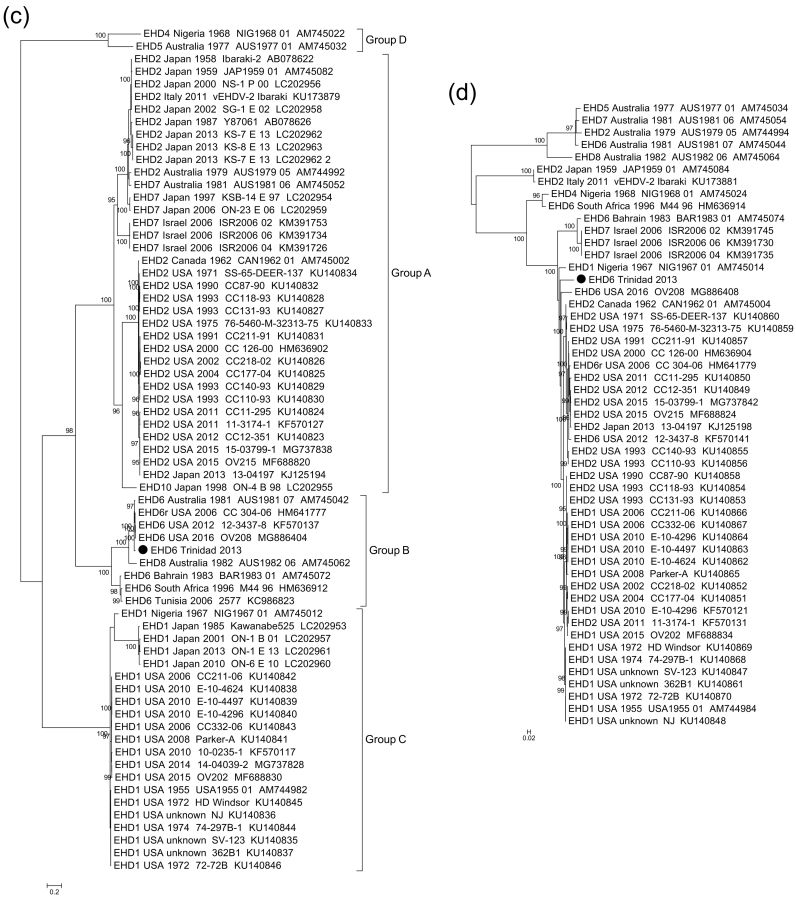
Phylogenetic trees were constructed for the coding regions of EHDV a) VP2 protein (segment-2), b) VP4 protein (segment 4), c) VP5 protein (segment 6) and d) NS2 protein (segment 8). Phylogenetic trees were constructed using IQ-Tree software (Nguyen et al., 2015) and the reliability of each tree was estimated by ultrafast bootstrap (Minh et al., 2013) analysis of 1000 replicates (bootstrap values of <95% are not shown). Capital letters A, B C and D correspond with four serological groups of EHDV.

Based on the phylogenetic analysis of segment 2 (VP2) and segment 6 (VP5), EHD6/Trinidad/2013 virus isolate clustered, with ≥95% ultrafast bootstrap support, into the serological group B which contains closely-related EHDV strains such as EHD6/AUS/1981/07 and the EHDV-6 reassortants from the USA: 2006 CC304–06, 2012 12–3437-8 and 2016 OV208 (Fig. 2a&2c). Phylogenetic analysis of EHDV segment 4/VP4 sequences demonstrated that the EHD6/Trinidad/2013 VP4 belongs to the eastern group, with the closest relative being EHD2/AUS/1979/05, rather than EHD6/AUS/1981/07 (Fig. 2b). In contrast, the phylogenetic analysis of segment 8/NS2 demonstrates that the EHD6/Trinidad/2013 NS2 is more closely related to the western EHDV strains from the USA, rather than the eastern strains from Australia (Fig. 2d).

## 4 Discussion

There is clear evidence that Orbivirus reassortment occurs frequently when a single cell is co-infected with at least two different strains (Allison et al., 2012; Batten et al., 2008; Nomikou et al., 2015; Samal et al., 1987). Reassortment is known to play an important role in the evolution of BTV (Nomikou et al., 2015), so it can be assumed that the same is true for EHDV. Field reassortment of EHDV-6 has been reported previously in North America (Allison et al., 2010), however, due to the limited amount of EHDV sequence data currently available, it is not possible to determine the extent of EHDV reassortment that is occurring in the field. At the time of writing, 51 complete genome sequences of EHDV were available through GenBank, of which 66% represent EHDV serotypes 1, 2 and 6 from the USA. This is much fewer compared to the over 400 complete genomes available for BTV (http://btv.glue.cvr.ac.uk/#/home). Despite the recent EHDV-6 outbreaks in beef and dairy cattle in Israel (Golender et al., 2017) and Japan (Kamomae et al., 2018) during 2015, and the many EHDV-6 outbreaks observed in Mediterranean countries, there are currently only six complete genomes of EHDV-6 available in GenBank. Three of these sequences are from EHDV-6 isolates from the 1980s and 1990s (AUS1981/07, BAR1983/01, and M44/96), and the remaining three are from the USA (CC 304-06, 12-3437-8, OV208).

In this study, we performed full genome sequencing of an EHDV-6 isolated from cattle in Trinidad and compared the full genome with available published genomes. The closest relative to EHD6/Trinidad/2013 virus was revealed to be an Australian EHDV-6 strain (EHD6/AUS1981/07). Eight out of the 10 segments showed a high degree of identity (97.2–97.9%) between these two viruses. This Australian strain was therefore identified as the major parental strain of the Trinidad virus. Other strains with equally high levels of VP2 homology with this Australian major parental strain have previously been reported in the Caribbean region, in Martinique and Guadeloupe in 2010 (Viarouge et al., 2014), however, full genome sequencing was not carried out on these viruses. In our previous study based on VP2 sequences, Bayesian coalescent analysis supported the possible incursion of EHDV-6 from Australia to the Americas and Caribbean regions and estimated that the divergence from the Australian prototype occurred ca. 1966 (Brown-Joseph et al., 2019). A reassortant strain of EHDV-6 Indiana was also detected in the USA, in which segments 2 and 6 originated from the same parent Australian strain (EHD6/AUS1981/07), however the remaining 8 segments of this USA virus originated from a local North American EHDV-2 from Alberta (Allison et al., 2010). Following the identification of this virulent EHD-6 reasssortant virus in the USA in 2007, questions have remained about the origin of the Australian parent strain and how it may have arrived in the USA.

The EHD6/Trinidad/2013 virus contained two reassorted segments, segments 4 encoding the VP4 protein and segment 8 encoding the NS2 protein. For segment 4, the highest percent identity (96.5%) was observed with the segment 4 of an Australian EHDV-2 virus (EHD2/AUS/1979/05), although the nucleotide identity between EHD6/Trinidad/2013 and EHD6/AUS/1981/07 was also quite high at 92.4%. These two Australian strains (EHDV-2 and EHDV-6) were identified in 1979 and 1981, so it is possible that they were co-circulating. It is therefore likely that the reassortment of segment 4 seen in the Trinidad virus occurred in Australia, at the time that these two viruses were co-circulating. Although EHDV-2 has been reported to be present on the island of Guadeloupe in 2011, the VP2 of this virus clustered with EHDV-2 strains from the USA, rather than from Australia (Viarouge et al., 2014). Although nine segments of the EHDV-6 Trinidad strain have been identified to be closely related to two Australian EHDV strains that were identified 30 years ago, the order of events describing how these segments found themselves in EHDVs circulating in North America and Trinidad remains unclear.

The reassortment of segment 8 in EHD6/Trinidad/2013 virus was highly supported by six independent methods (GENECONV, BOOTSCAN, Maximum Chi Square, CHIMAERA, SISCAN and 3SEQ) when the 31 full genome sequences of EHDV were analysed using the RDP. The RDP analysis did not however identify a minor parent for segment 8 of the EHDV/Trinidad/2013 virus from the 31 full genome sequences of EHDV that were analysed. Due to lack of sequencing data enabling the identification of the minor parent, the closest relative was selected as the plausible minor parent for segment 8 of the EHD6/Trinidad/2013 virus as an EHDV-1 strain from the USA (EHD1/USA/1955/01). Due to a lack of active epidemiological surveillance and the often subclinical nature of EHD, many EHDV serotypes/reassortants may remain undetected in the Caribbean, South America and elsewhere in the world. It is therefore possible that the minor parent strain for NS2 could be circulating, yet unidentified, in Trinidad. Although EHDV-1 was not identified to be circulating at the single location where the cattle were sampled in this study, it is possible that this serotype is present elsewhere on the island. A more extensive sampling exercise across the whole of the island would be necessary to see if this was the case. Alternatively, the Trinidad EHDV-6 could have received its NS2 by reassorting with a locally circulating virus in a neighbouring South American country, with the reasssorted virus then being transported to Trinidad. Co-circulation of EHDV-1 and EHDV-6 was reported in young cattle in French Guiana in the summer of 2011 (Viarouge et al., 2014). The proximity of French Guiana, Guyana and Venezuela on the South American mainland makes it highly likely that these two EHDV serotypes are circulating in all three countries. As Trinidad lies only 10 km off the North East coast of Venezuela, and there is a history of illegal movement of cattle between the two countries, it is very possible that EHD viruses have entered Trinidad through this trading route. Additionally, *Culicoides* vectors infected with EHDV could have transported the virus by boat or by wind between the two countries. Unfortunately, the full genomes of EHDV-1 and EHDV-6 from French Guiana were not available, so comparison of the segment 8 sequences between the Trinidad and French Guiana viruses was not possible.

This data strongly suggests that the EHD6/Trinidad/2013 virus had an Australian origin, receiving 9 of its 10 segments from two viruses circulating in Australia. This reassortant virus then likely came to the Americas, where it received its segment 8 from a locally circulating, as yet unknown, EHDV strain. This virus then may have gained entry into the USA, where it further reassorted with a known locally circulating EHDV-2 strain, giving rise to the EHDV-6 Indiana strain, with segments 2 and 6 originating from the EHDV-6 Trinidad strain and its remaining 8 segments originating from the locally circulating EHDV-2 strain. This study therefore identifies, for the first time, the likely minor parent virus (EHD6/Trinidad/2013) of the EHDV-6 Indiana strain currently circulating in the USA.

Similar to BTV, the reasons behind the wide range of virulence observed between different EHDV strains within the same serotype is not fully understood. These differences in virulence could in part be attributed to different susceptibilities of hosts and/or differences in the competence of the local *Culicoides* vectors. Critically however, when trying to understand more about the mechanisms behind viral virulence, it is vital to carry out full genome sequencing of all 10 segments of the virus, in order to know whether the virus has undergone reasssortment and to identify the parent strains of the reassorted segments. Knowing which segments have reassorted, as well as the virulence of the parent viruses, could provide evidence as to which of the segments are playing a role in virulence.

The EHD6/Trinidad/2013 double-reassortant did not cause clinical signs of disease in the group of naïve Holstein and Jersey cattle in this study. This was also the case in cattle infected with its major parental virus from Australia, EHD6/AUS/1981/07 (St. George et al., 1983). Interestingly, cattle in the USA infected with the reassortant EHDV-6 Indiana strain showed clinical signs (Allison et al., 2010). Following the identification of this EHDV-6 Indiana (Anbalagan et al., 2014), a sudden increase of disease caused by EHDV-6 was reported across Nebraska, South Dakota, Michigan and Missouri in domestic cattle and white-tailed deer (Stevens et al., 2015). This points towards the possible emergence of this virulent virus at this time, which may have coincided with a reassortment event between the avirulent Australian-derived EHDV-6 reassortant strain and the virulent locally circulating EHDV-2 strain from the USA (Ruder et al., 2017). This agrees with previous findings showing VP2 and NS3 to be the main determinants of BTV-8 pathogenicity, however other viral proteins such as VP1, VP4, VP5, VP6 and VP7 were shown to also influence virulence (Janowicz et al., 2015).

To better understand the origin of emerging EHDV strains, their transmission and their potential for generating reassortants, full genome sequencing is required. This study shows how full genome sequencing of Orbiviruses can enhance the ability to support epidemiological investigations on tracing viral incursions from distinct geographical locations. In addition, we also identified ‘missing links’ in the epidemiology of EHDV as the donor virus of segment-8 in EHDV-6 from Trinidad still reminds unknown. Improved pipelines for full genome sequence analysis, along with cheaper and more accessible sequencing platforms, should lead to an increase in published EHDV sequences, making the tracing of incursions more accurate and more representative to different regions of the world. It will also enable the direct comparison of reassortment frequencies between EHDV and BTV. Although a reverse genetics system has been developed for EHDV (Yang et al., 2015), the influence of individual segments on virulence has not been studied. This presents an interesting opportunity for further research.

## 5 Conclusions

In this study, a double-reassortant EHDV-6 strain, with an EHD6/AUS/1981/07 backbone (8 segments) and minor parental strains from EHD2/AUS/1979/05 (segment 4) and an unknown EHDV strain (segment 8), was identified to be circulating asymptomatically in cattle in Trinidad, West Indies. These data provide evidence that the minor parent of EHDV-6 Indiana is circulating in the Caribbean region and likely spread northwards from the Caribbean, or South America, to North America, before reassorting with a locally circulating EHDV-2 strain in the USA. This addresses the long-unanswered question related to the origin of the Australian parent strain of EHDV Indiana and how it may have arrived in the USA.

The following are the supplementary data related to this article.

Table 1 Full-genome EHDV reference sequences. Table 1
